# Sarcopenia Predicts Early Dose-Limiting Toxicities and Pharmacokinetics of Sorafenib in Patients with Hepatocellular Carcinoma

**DOI:** 10.1371/journal.pone.0037563

**Published:** 2012-05-30

**Authors:** Olivier Mir, Romain Coriat, Benoit Blanchet, Jean-Philippe Durand, Pascaline Boudou-Rouquette, Judith Michels, Stanislas Ropert, Michel Vidal, Stanislas Pol, Stanislas Chaussade, François Goldwasser

**Affiliations:** 1 Centre for Research on Angiogenesis Inhibitors (CERIA), Department of Medical Oncology, Cochin Teaching Hospital, AP-HP, Université Paris Descartes, Sorbonne Paris Cité, Paris, France; 2 Department of Clinical Pharmacology, Cochin Teaching Hospital, AP-HP, Université Paris Descartes, Sorbonne Paris Cité, Paris, France; 3 Department of Gastro-Enterology, Cochin Teaching Hospital, AP-HP, Université Paris Descartes, Sorbonne Paris Cité, Paris, France; 4 Laboratory of Pharmacology and Toxicology, Cochin Teaching Hospital, AP-HP, Université Paris Descartes, Sorbonne Paris Cité, Paris, France; 5 Department of Hepatology and INSERM U1016, Cochin Teaching Hospital, AP-HP, Université Paris Descartes, Sorbonne Paris Cité, Paris, France; Kanazawa University, Japan

## Abstract

**Background:**

Sorafenib induces frequent dose limiting toxicities (DLT) in patients with advanced hepatocellular carcinoma (HCC). Sarcopenia has been associated with poor performance status and shortened survival in cancer patients.

**Patients and Methods:**

The characteristics of Child Pugh A cirrhotic patients with HCC receiving sorafenib in our institution were retrospectively analyzed. Sorafenib plasma concentrations were determined at each visit. Toxicities were recorded during the first month of treatment, and sarcopenia was determined from baseline CT-scans.

**Results:**

Forty patients (30 males) were included. Eleven (27.5%) were sarcopenic. Eighteen patients (45%) experienced a DLT during the first month of treatment. Sarcopenic patients experienced significantly more DLTs than non-sarcopenic patients did (82% versus 31%, p = 0.005). Grade 3 diarrhea was significantly more frequent in sarcopenic patients than in non-sarcopenic patients (45.5% versus 6.9%, p = 0.01), but not grade 3 hand foot syndrome reaction (9% versus 17.2%, p = 1). On day 28, median sorafenib AUC (n = 17) was significantly higher in sarcopenic patients (102.4 mg/l.h versus 53.7 mg/l.h, p = 0.013).

**Conclusions:**

Among cirrhotic Child Pugh A patients with advanced HCC, sarcopenia predicts sorafenib exposure and the occurrence of DLT within the first month of treatment.

## Introduction

Hepatocellular carcinoma (HCC) accounts for more than 24,000 new cases per year in the United States, and causes approximately 19,000 deaths yearly [1]. The incidence of HCC is increasing in Western countries, and HCC is diagnosed at advanced stages in up to 60% of all patients [2].

Sorafenib (Nexavar; Bayer Healthcare, Wayne, NJ) is an orally active multi–kinase inhibitor that targets BRAF, RET, PDGFR-β, VEGFR-1, and VEGFR-2 [3]. At a dose of 400 mg bid, sorafenib displays clinical activity in patients with advanced HCC [4]. Sorafenib-induced toxicities (diarrhea, hand-foot syndrome, rash, fatigue and hypertension) may limit patient's ability to receive full-dose treatment, and resulted in dose reductions in 26% and treatment termination in 44% of patients included in the pivotal phase III trial [4].

Little is known on factors predicting sorafenib toxicity. Recently, the condition of low muscle mass named sarcopenia [5], [6] was identified as a significant predictor of toxicity in patients with metastatic renal cancer treated with sorafenib 400 mg bid [7]. Sarcopenia has been studied mainly in geriatric populations [5], but also more recently in cancer patients, in whom sex-specific cut-offs were determined, based on mortality risks [8]. Sarcopenia and overall lean body mass may represent an occult condition in cancer patients with normal or even high body mass index (BMI). Sarcopenia has been associated with poor PS, capecitabine and epirubicin toxicity, and shortened survival in cancer patients [8], [9], [10].

The purpose of the present study was to investigate whether sarcopenia could predict the occurrence of early dose-limiting toxicities (DLT) in HCC patients treated with sorafenib. We hypothesized that an increased toxicity, as well as a higher drug exposure, would be observed in sarcopenic HCC patients.

## Materials and Methods

### Participants

We performed a retrospective electronical medical record review of consecutive patients with advanced HCC treated with sorafenib in our institution from June 2007 to December 2010. From October 2008, a population pharmacokinetics study including patients receiving sorafenib for various malignancies was initiated [11]. The pharmacokinetic data of patients with HCC were extracted for the present study.

### Ethics

All patients provided written informed consent, and the study was approved by the local ethics board according to good clinical practice and applicable laws, and the declaration of Helsinki.

### Treatment, toxicity and activity assessment

Adult patients with HCC received sorafenib at a starting dose of 200 mg bid or 400 mg bid according to their ECOG PS and co-morbidities, at the discretion of the treating physician, as described by other authors [12]. Patients were treated in an outpatient setting, and toxicity was assessed at visits on days 14 and 28 after the initiation of sorafenib (or before if clinically indicated), then monthly.

Sorafenib dose was reduced to 200 mg bid in the case of severe toxicity (grade 3 or 4 toxicity according to the NCI-CTC v3.0), except for patients with grade 3 hypertension in whom anti-hypertensive drugs were introduced according to current guidelines [13]. If toxicity was not thereby resolved, treatment was terminated, and if so, patients were returned to the initially scheduled dose.

**Figure 1:**
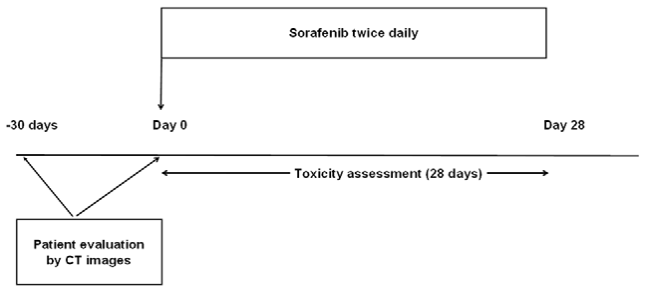
Study timelines.

A DLT was defined as any toxicity leading to dose reduction, temporary or permanent or discontinuation of treatment. Following the design of a previous study [9], only DLTs occurring during the first month of treatment were examined for the present analysis (Figure 1).

Treatment activity was assessed every three months by CT-scan, or before if clinically indicated, according to RECIST v1.0.

**Figure 2:**
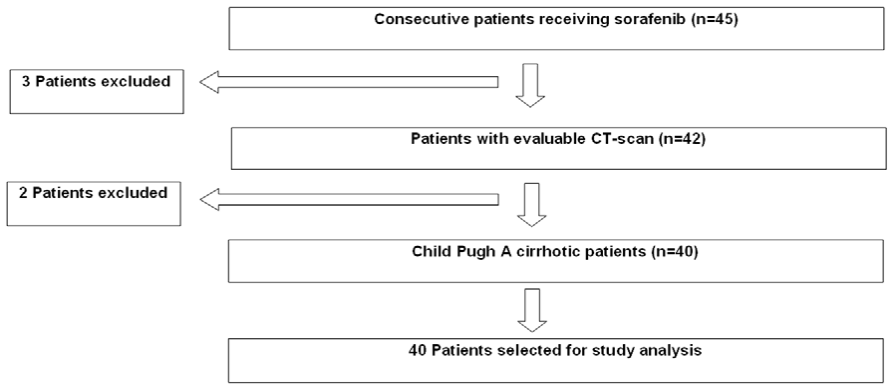
Patients selection for the present study.

Patients' characteristics are described in Table 1. Patients presented with a broad range of LBM. Therefore, the administered dose of sorafenib was markedly variable when expressed per kg of LBM, ranging from 3.8 to 16.8 mg/kg LBM bid. Eleven (27.5%) of 40 patients had sarcopenia (10 men and 1 woman).

**Table 1:** Baseline characteristics of cirrhotic patients treated with sorafenib.

	*Males*	*Females*	*Total*
*Patients*	*30*	*10*	*40*
Age, years: median (range)	63.5 (32–79)	60 (41–78)	62.5 (32–79)
ECOG PS, n (%)			
0	8 (27)	4 (40)	12 (30)
1	13 (43)	5 (50)	18 (45)
≥2	9 (30)	1 (10)	10 (25)
Specific metastatic sites, n:			
Lung	7	5	12
Liver	25	9	34
Other	7	4	11
Duration of disease, months: median (range)	6.8 (0.4–36.8)	12.4 (2.7–42.7)	7.4 (0.4–42.7)
Etiology of Cirrhosis; n:			
Hepatitis C	6	3	9
Hepatitis B	9	1	10
Alcohol	5	0	5
Other	10	6	16
Alpha-foetoprotein, ng/mL: median (range)	3500 (10–272400)	2750 (2–178600)	3380 (2–272400)
Weight (kg): median (range)	69 (47–93)	63 (40–98)	68.5 (40–98)
BMI: median (range)	23.7 (17.3–31.8)	24.4 (18.0–37.3)	23.9 (17.3–37.3)
Underweight (BMI<18.5), n (%)	1 (3)	1 (10)	2 (5)
Normal weight (18.5<BMI<24.9), n (%)	19 (63)	5 (50)	24 (60)
Overweight (25<BMI<29.9), n (%)	8 (27)	2 (20)	10 (25)
Obese (BMI>30), n (%)	2 (7)	2 (20)	4 (10)
Estimated LBM (a) (kg): median (range)	52.3 (23.9–64.1)	33.1 (28.0–39.5)	48.3 (23.9–64.1)
Skeletal muscle L3 area (b) (cm^2^): median (range)	168 (74–208)	104 (87–126)	155 (74–208)
Skeletal muscle L3 index (c) (cm^2^/m^2^): median (range)	57.2 (28.2–70.2)	40.7 (33.3–47.3)	53.9 (28.2–70.2)
% Sarcopenic	33.3	10.0	27.5
Adipose tissue L3 area (b)(cm^2^): median (range)	318.8 (102.6–847.2)	349.6 (48.3–701.5)	334.2 (48.3–847.2)
Adipose tissue L3 index (c)(cm^2^/m^2^): median (range)	109.4 (31.7–289.7)	137.4 (21.8–267.3)	119.9 (21.8–289.7)

*ECOG, Eastern Cooperative Oncology Group Criteria Performance Status; BMI, Body Mass Index (weight/height^2^).*

(a) Calculated from the regression equation: whole lean body mass (kg) = 0.30×[[skeletal muscle at L3 using CT (cm^2^)]+6.06]

(b) Total tissue sectional area at L3 (cm^2^)

(c) Lumbar tissue index: area/height (cm^2^/m^2^).

### Anthropometric measurements

Weight was measured with a medical balance beam scale and height was measured with a stadiometer. Body mass index (BMI) was calculated [weight (kg)/height (m^2^)] and the World Health Organization (WHO) categories were used: underweight, BMI<18.5; normal, 18.5<BMI<24.9; overweight, 25<BMI<29.9; obesity, BMI>30. Body surface area (BSA) was calculated using the Mosteller formula: body-surface area (m^2^) = ([height (cm)×weight (kg)]/3600)^1/2^.

### Image analysis

Body composition was evaluated using CT-scan, as previously described [14], [15]. Regional adipose tissues (visceral and subcutaneous) and muscle tissues were assessed on CT images, which had been performed for diagnostic and follow-up purposes. Images taken within 30 days before initiation of sorafenib were included (Figure 1). Images were analyzed using ImageJ software v1.42q (National Institutes of Health, http://rsb.info.nih.gov/ij).

The third lumbar vertebra (L3) was chosen as a standard landmark, as previously described [7]. Muscles were identified based on their anatomic features, and the structure of those specific muscles were quantified based on pre-established thresholds of Hounsfield units (−29 to +150) of skeletal muscle tissue [15]. Cross-sectional areas (cm^2^) of the sum of all of these muscles were computed for each image, and the mean value for two consecutive images was computed for each patient.

Total lumbar skeletal muscle cross-sectional area (cm^2^) and total lumbar adipose tissue area (cm^2^), are linearly related to whole-body muscle and adipose tissue mass [16], [17]. These values were normalized for stature as is conventional for BMI and body composition components [8], [16] and expressed in units of cm^2^/m^2^. The sex-specific cut-offs for sarcopenia [8] determined in patients with digestive malignancies (55.4 cm^2^/m^2^ for males and 38.9 cm^2^/m^2^ for females) were used.

Total lean body mass (LBM) was estimated from muscle cross-sectional areas as described by Mourtzakis et al. [16]: LBM (kg) = 0.30×[skeletal muscle at L3 using CT (cm^2^)]+6.06.

**Table 2:** Comparisons between patients with and without sarcopenia.

	*Sarcopenic (n = 11)*	*Non-sarcopenic (n = 29)*	*p*
**Sorafenib starting dose: n (%)**			
200 mg bid	6 (54.5)	5 (17.2)	**0.04**
400 mg bid (standard dose)	5 (46.5)	24 (82.8)	
**ECOG PS: n (%)**			
0–1	8	22	1
2	3	7	
**Characteristics: median (range)**			
Age	66 (42–78)	62 (32–79)	0.55
Weight (kg)	71 (61–98)	68 (40–93)	0.21
Height (m)	1.70 (1.53–1.91)	1.70 (1.49–1.80)	0.80
BMI (kg/m^2^)	23.8 (18.7–37.3)	23.9 (17.3–35.0)	0.90
BSA (m^2^)	1.83 (1.61–2.15)	1.78 (1.29–2.13)	0.09
Albuminemia at baseline (g/l)	34 (28–39)	38.5 (28–44)	**0.004**
CRP at baseline (mg/l)	9.3 (2.2–49)	7.8 (1–104)	0.80
Lumbar skeletal muscle index (cm^2^/m^2^)^a^	34.6 (28.2–51.7)	57.3 (38.9–70.1)	**<0.001**
Whole body lean body mass (kg)	32.9 (23.9–58.4)	52.3 (28.5–64.1)	**0.003**
Sorafenib dose (mg) per kg of LBM, bid	6.9 (4.6–16.8)	7.5 (3.8–14.0)	0.70
**DLT from day 1 to day 28: n (%)**			
Present	9 (81.8)	9 (31.0)	**0.005**
Absent	2 (19.2)	20 (69.0)	
**Toxicity prevalence from day 1 to day 28: n (%)**			
Hand-foot syndrome, all grades	3 (27.3%)	14 (48.3%)	0.30
Grade 3 hand-foot syndrome	1 (9.0%)	5 (17.2%)	1
Diarrhea, all grades	6 (54.5%)	8 (27.6%)	0.15
Grade 3 diarrhea	5 (45.5%)	2 (6.9%)	**0.01**
Asthenia, all grades	5 (45.5%)	10 (34.5%)	0.72
Grade 3 asthenia	2 (27.3%)	2 (6.7%)	0.30
Hypertension, all grades	3 (27.3%)	10 (34.5%)	1
Grade 3 hypertension^b^	0	3 (10.3%)	0.55
**Sorafenib dose-adjusted AUC on day 28, mg/l.h:** median (range)	102.4 (48.0–137.8)	53.7 (24.5–74.5)	**0.013**

*SD, standard deviation; ECOG, Eastern Cooperative Oncology Group Criteria Performance Status; BMI, Body Mass Index [weight(kg)/height(m)^2^]; BSA, Body Surface Area.*

(d) Calculated from regression equation: whole lean body mass (kg) = 0.30×[[skeletal muscle at L3 using CT (cm^2^)]+6.06].

(b) Hypertension was not considered as a dose limiting toxicity.

**Figure 3:**
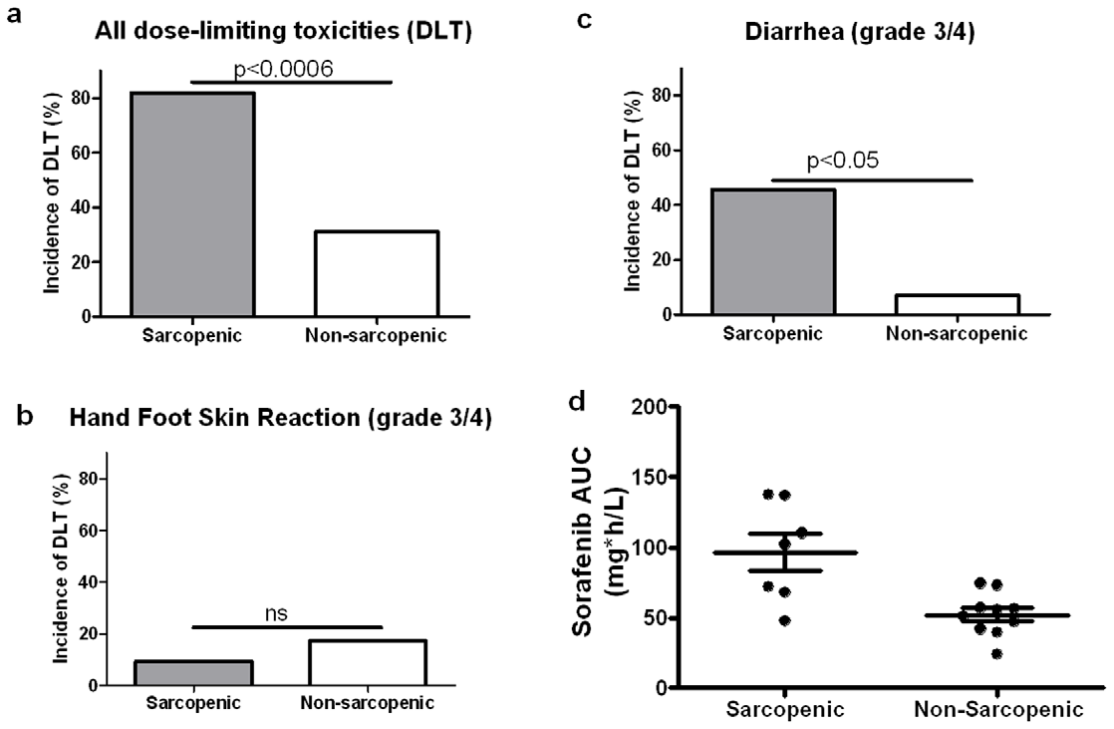
Prevalence of dose-limiting toxicities and estimated sorafenib AUC in sarcopenic and non-sarcopenic patients.

### Pharmacokinetic measurements

On each visit, blood samples were drawn to determine sorafenib plasma concentrations. Sorafenib concentration determination was conducted using a previously described high-performance liquid chromatography method [18]. The accuracy, within-assay and between assay precision of this method were 96.9–104.0%, 3.4–6.2% and 7.6–9.9%, respectively. A specific bayesian estimator developed in our institution [11] allowed estimating individual sorafenib area under the plasma concentration–time curve from 0 to 12 hours (AUC). A 1-compartment model with saturated absorption, first-order intestinal loss and linear elimination was implemented in the ADAPT II software package, then used to estimate sorafenib exposure (AUC).

### Statistical analysis

Prevalence of toxicity was compared by Fisher's exact test. The Wilcoxon test was used for comparison of continuous variables, and the Mann-Whitney U-test was used for comparisons between AUCs. AUCs were normalized to a dose of 400 mg sorafenib twice daily to investigate their relation with BMI, LBM or adipose index. Dose-standardized AUCs were also used to compare the drug exposition between non- and sarcopenic patients. The impact of baseline parameters, including sarcopenia, on the occurrence of DLT during the first month of treatment was evaluated by multiple regression with 3000 bootstrap iterations. All p values were two-sided, and the level of significance was p<0.05.

Progression-free survival (PFS) and overall survival (OS) were measured from the date of first treatment administration to the date of disease progression or death for the former, and the date of death for the latter. Kaplan-Meier estimates of the distribution of times from baseline to outcome were computed, and the groups were compared using the log-rank test. Calculations were performed with NCSS™ 2007 software (NCSS, Kaysville, UT).

## Results

### Patients

Among 45 patients received sorafenib for advanced HCC from June 2007 to December 2010, 42 had computerized tomography (CT) images that met criteria for analysis. Three patients were excluded from the investigation because they had either no CT scans on record (n = 2), or a CT scan >30 days from treatment initiation (n = 1). Finally, two additional patients with Child B cirrhosis were also excluded. Overall, 40 patients with advanced HCC and Child A cirrhosis were selected for the present analysis (Figure 2), and AUCs on day 28 were available for 17 (43%) patients.

### Sorafenib toxicity

Eighteen patients (45%) experienced a DLT during the first month of treatment (Table 2). The dose was reduced to 200 mg bid for the 12 patients who had been started on sorafenib at a dose of 400 mg bid. In the remaining 6 patients, who had been started on sorafenib 200 mg bid, the treatment was discontinued.

DLTs included grade 3 diarrhea in 7 cases, grade 3 hand-foot syndrome in 6 cases, and grade 4 upper GI bleeding in 1 case. The remaining four patients had multiple, simultaneous grade 2 toxicities, with grade 3 fatigue. Grade 3 hypertension occurred in 3 patients (all non-sarcopenic), but was not considered as dose-limiting since it was controlled by the introduction of oral anti-hypertensive agents, without modification of sorafenib dosing.

The comparison between sarcopenic and non-sarcopenic patients is summarized in Table 2. Six of 11 sarcopenic patients had been started on sorafenib 200 mg bid, a higher proportion than that observed in non-sarcopenic patients (54.5% vs. 17.2%, p = 0.04). However, no difference was observed regarding sorafenib dose/kg of LBM, age, height, weight, BMI and BSA. As expected, significant differences in serum albumin, lumbar skeletal muscle index and LBM were seen between sarcopenic and non-sarcopenic patients.

Overall, patients with sarcopenia presented with a higher prevalence of DLT (81.8% or 9/11) compared with non-sarcopenic patients (31.0% or 9/29, p = 0.005; Table 2, Figure 3a). When considering each toxicity separately, only grade 3 diarrhea was significantly more prevalent in sarcopenic patients (5/11 patients or 45.5% vs. 2/29 patients or 6.9%, p = 0.01, Figure 3b). No significant difference was found for other toxicities, especially hand-foot skin reaction (Figure 3c). By multivariate analysis, only sarcopenia was an independently correlated with the occurrence of DLT (p = 0.03).

### Sorafenib pharmacokinetics

The median calculated dose-adjusted AUC on day 28 was higher in sarcopenic patients than in non-sarcopenic patients (median: 102.4 mg/l.h, range: 48.0–137.8 vs. 53.7 mg/l.h, range: 24.5–74.5, respectively, p = 0.013, Figure 3d). No correlation was found between AUC and BMI, LBM or adipose index (p = 0.34, 0.11 and 0.23, respectively). Patients who experienced a DLT during the first 28 days of treatment had higher AUCs on day 28 (median: 106.4 mg/l.h, range 48–177.8 vs 56.7 mg/l.h, range 24.5–136.7), although this difference was not statistically significant (p = 0.09). As well, no statistical difference was found between AUC on day 28 in patients with or without grade 3 diarrhea (median: 102.4 mg/l.h, range 48–137.8 vs. 56.9 mg/l.h, range 24.5–136.7, respectively, p = 0.10).

### Survival analysis

**Figure 4:**
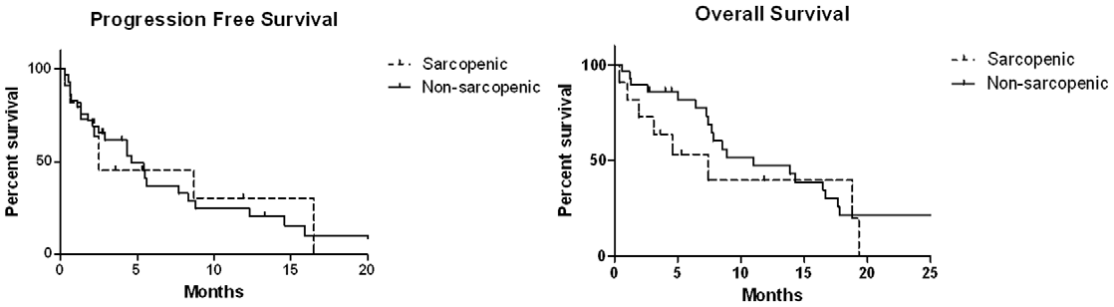
Progression-free and overall survival in sarcopenic (dot line) and non-sarcopenic (solid line) HCC patients receiving sorafenib.

The median PFS and OS for the study population (n = 40) were 4.6 (95%CI: 2.5–7.7) and 8.9 months (95%CI: 7.4–16.5), respectively. No significant differences were observed between non-sarcopenic and sarcopenic patients regarding median PFS [4.6 (95%CI: 2.5–7.7) vs. 2.5 months (95%CI: 1.3–16.1), respectively, p = 0.94] and median OS [11.0 (95%CI: 7.7–16.5) vs. 7.4 months (95%CI: 1.9–19.3), respectively, p = 0.28] (Figure 4).

## Discussion

The present retrospective analysis is the first study evaluating relationships between body composition and chemotherapy toxicity and pharmacokinetics in patients with HCC. In a daily practice population of 40 patients with HCC and Child A cirrhosis, we found a proportion of 27.5% of patients with sarcopenia, a proportion similar to that observed in other series [19].

Sarcopenic patients received more frequently an initial dose of sorafenib of 200 mg bid based on the clinical judgement of the treating physician, a finding consistent with the knowledge that sarcopenia has been associated with poor PS in cancer patients [8]. Interestingly, despite the apparent heterogeneity in sorafenib dosing between sarcopenic and non-sarcopenic patients, the doses of sorafenib/kg of LBM were identical in both groups.

During the first month of treatment, sarcopenic patients experienced significantly more DLTs, mainly grade 3 diarrhea. DLTs were frequent in our patients, probably because they represents a daily practice population, more frail and more prone to develop severe toxicity than patients treated in clinical trials.

Sarcopenic patients had higher sorafenib AUC on day 28 than non-sarcopenic patients, but we found no significant correlation between AUC and the occurrence of DLT or grade 3 diarrhea, although a trend was observed (p = 0.09 and p = 0.10, respectively). This negative result may be due to the small number of patients with pharmacokinetic data, since routine monitoring of sorafenib plasma concentrations has recently started in our institution. We therefore hypothesize that the ongoing collection of pharmacokinetic data in our institution could allow the demonstration of a relationship between increased exposure and the occurrence of severe diarrhea. Of note, phase I trials have failed to demonstrate a clear correlation between sorafenib exposure and other toxicities [20].

Several hypotheses accounting for increased exposure and increased toxicity in sarcopenic patients with HCC can be raised.

Firstly, sarcopenia might result in alterations in the distribution, metabolism and clearance of anticancer drugs. Prado et al. [10] have recently evidenced a critical role for LBM in the pharmacokinetics of epirubicin. In line with Prado's hypothesis regarding epirubicin, it is of interest to underscore that sorafenib is highly protein-bound (>99%, mainly to albumin). Binding might also occur in lean body tissues, and not exclusively to serum albumin. Hence, patients with sarcopenia would have high intra-tissular exposure to unbound sorafenib. A potential confounding factor could be hypoalbuminemia, which was more prevalent in sarcopenic patients in our study. The pharmacokinetics of sorafenib in patients with low albuminemia remains unknown. Miller et al. [21] investigated total and free sorafenib exposure in cancer patients with varying degrees of hepatic dysfunction receiving a single-dose of sorafenib 400 mg. In contradiction to *a priori* hypothesis, sorafenib unbound fraction in a cohort including patients with severe hypoalbuminemia (<25 g/L) were not significantly different from those measured in the cohort with normal hepatic function. However, the clinical relevance of this study using a single-dose of sorafenib 400 mg is limited, because it does not provide information on sorafenib pharmacokinetics and toxicity when the steady-state has been reached. Hence, the consequences of hypoalbuminemia in patients receiving sorafenib remain to determine.

Secondly, Antoun et al. [7] found that sarcopenia and low BMI could predict DLT (at any time during the treatment) in patients with advanced renal cancer. The authors hypothesized that in patients with low BMI and sarcopenia, sorafenib flat-dosing (400 mg bid) could result in increased exposure, and thereby in excessive toxicity. Our results confirm this hypothesis, although we found no relationship between BMI and the occurrence of sorafenib-induced DLT in patients with HCC. This may be due to the fact that patients with Child-Pugh A cirrhosis might develop lower limb oedema and mild ascites, leading to erroneously normal calculated BMI. Antoun et al. [7] underscored that sarcopenic patients have an increased propensity for nosocomial infections and other complications in hospital [22], a possible reflect of a global frailty that might include vulnerability to sorafenib toxicity.

Thirdly, systemic inflammation underlies sarcopenia [6], and might play a role in the occurrence of DLT. Indeed, inflammation has a negative impact on the activity of CYP3A4 [23], one of the enzymes involved in the metabolism of sorafenib. Hence, sarcopenic patients with high baseline CRP levels could experience high exposure to sorafenib due to low CYP3A4 activity, and subsequently excessive toxicity. In the present study, we found no difference in baseline CRP levels between sarcopenic and non-sarcopenic patients. However, the impact of sarcopenia on CYP3A4 activity deserves further investigations, including phenotypic and genotypic testing.

The limitations of the present study include its retrospective nature and the small number of patients. Further prospective studies including a PK/PD approach are needed to validate these results in larger populations of patients. Our results also pinpoint that the evaluation of LBM based on CT-scan analysis is worth being investigated in patients treated with sorafenib, rather than other clinical parameters such as body weight, BSA or BMI.

In conclusion, our results highlight the emerging role of sarcopenia assessment to improve the anticipation of sorafenib-related toxicities, opening gates to drug dosing individualization in patients with advanced HCC. This concept warrants validation in further prospective studies evaluating toxicity after drug dosing based on pre-treatment evaluation of sarcopenia.
